# The Cleaner XT™ Device as an Endovascular Adjunct for Pharmacomechanical Thrombolysis of Thrombosed Arteriovenous Fistulas and Grafts

**DOI:** 10.3400/avd.oa.20-00046

**Published:** 2020-12-25

**Authors:** Khian Wan Sarah Joy Huan, Chieh Suai Tan, Deborah Chua, Charyl Jia Qi Yap, Ru Yu Tan, Tze Tec Chong, Tjun Yip Tang

**Affiliations:** 1 Department of Vascular Surgery, Singapore General Hospital, Singapore; 2 Department of Renal Medicine, Singapore General Hospital, Singapore; 3 Duke-NUS Medical School, National University of Singapore, Singapore

**Keywords:** CDT, thrombolysis, Cleaner XT™, primary patency, AVF

## Abstract

**Objective:** This paper documents our experience using the Cleaner XT™ device (Argon Medical Devices, Plano, TX, USA) for pharmacomechanical thrombolysis (PMT) of thrombosed haemodialysis arteriovenous grafts (AVG) and fistulas (AVF).

**Materials and Methods:** This was a retrospective case series (n=17) over six months at Singapore General Hospital. We evaluated demographics, procedural data, technical and procedural success, patency rates and complications.

**Results:** There were 8 (47%) males and the patients’ mean age was 66 (± 5.7) years. The mean age of AVF/AVG was 1605 (± 1099) days. All procedures were performed under local anaesthetic. PMT was performed within a mean time of 40 (±34.3) hours from the presentation. Technical, clinical and procedural success was 15/17 (88%). The thrombolysis agents used were tissue plasminogen activator (52.9%) and urokinase (41.2%). Mean primary patency time was 114 (± 116) days, with a 65% 1-month and 47% 3-month primary patency rates. The mean secondary patency time was 155 (±132) days, with 76% one-month and 65% three-month secondary patency rates, respectively. AVF rupture occurred in 3/17 (18%) cases but did not involve loss of the access circuit.

**Conclusion:** The Cleaner XT™ device is a safe, minimally invasive endovascular tool for PMT in thrombosed AVF/AVG, with relatively high success and low complication rates.

## Introduction

Haemodialysis can be performed via an arteriovenous fistula (AVF) or arteriovenous graft (AVG). These procedures can be prone to thrombosis because of progressive narrowing from neo-intimal hyperplasia and repeated trauma from cannulation, leading to loss of access if left untreated. This failure leads to significant morbidity and mortality in patients on hemodialysis.^1)^ The vascular access should be declotted promptly to avoid thrombus solidification and consequent loss of the arteriovenous access circuit.^2)^ This can be performed either through an open surgical or endovascular percutaneous thrombectomy. The latter is associated with lower morbidity and mortality as well as faster recovery, which is especially important in end-stage renal disease (ESRD) patients with multiple comorbidities.^3)^ Various endovascular techniques have been described, including catheter-directed thrombolysis (CDT) and mechanical thrombectomy. Such techniques are often employed in conjunction with treating the underlying pathology, usually stenosis, by balloon angioplasty.^4)^ Pharmacomechanical thrombolysis (PMT) was developed as a means of combining open and endovascular approaches with direct removal of a clot and adjunctive thrombolysis, achieving complete clot resolution with reduced thrombolytic dose, cost and treatment time.^5)^

PMT is an effective technique comparable to surgical thrombectomy for treating thrombosis in AVF and AVG.^6,7)^ Furthermore, its outcomes were comparable to that of CDT in deep vein thromboses in terms of patency rate, with a shorter length of stay, leading to lower costs.^8)^ There are a range of PMT devices available, with different mechanisms of action, broadly divided into two categories.^9)^

1. Hydrodynamic recirculation devices. The mechanism is based on the Venturi effect produced by high-speed saline jets, where the thrombus is sucked into the device and macerated. The products are then removed by an exhaust lumen in the device. An example would be the Angiojet Rheolytic System (Boston Scientific, Marlborough, MA, USA). This device has a 360-degree suction vortex, which may reduce the number of passes and the need for rotational positioning. It also functions isovolumetrically, which minimises the risk of unintentional hypovolemia during declotting.
2. Rotational recirculation devices. The clot is fragmented via generation of a hydrodynamic vortex, which is created by a high-speed rotating impeller or basket. An example would be the Arrow–Trerotola percutaneous thrombectomy device (Teleflex, Wayne, PA, USA), which is simple and inexpensive to manufacture but causes significant endothelial denudation in native vessels.^10)^

This study documents our experience using the Cleaner XT™ Rotational Thrombectomy System (Argon Medical Devices, Plano, TX, USA) for PMT of thrombosed AVG and AVF. We looked at its safety, efficacy and complications as well as the procedural tips we learnt from using the device.

## Materials and Methods

### Patient cohort

This is an investigator-initiated pilot single centre, single-arm retrospective case series study over six months (Dec 2018–July 2019) at Singapore General Hospital (SGH). The local Human Research Ethics Committee approved this study (CIRB ref: 2018/2557). SGH is the oldest restructured government hospital, situated in central Singapore and performs over 3000 AVG or AVF salvage procedures annually.

We used the Cleaner XT™ device with a thrombolytic agent to unblock 5 AVGs and 12 AVFs in 17 patients. All procedures were performed in the Interventional Nephrology Suite at SGH under local anaesthesia.

Patients with ESRD presenting with a thrombosed AVG or AVF were screened for eligibility. The preoperative diagnosis of a thrombosed non-functioning AVF/AVG was established clinically based on a lack of thrill or bruit or a duplex dependent diagnosis and the inability to have hemodialysis. Eligible patients were offered participation. All participants received an explanation of the potential benefits and risks of PMT using the Cleaner XT™ device before giving informed consent.

Inclusion criteria:
  • Age 21–85 years
  • Thrombosed AVG/AVF in the arm
Exclusion criteria:
  • Patient unable to provide informed consent
  • Sepsis or active infection
  • Recent intracranial bleed or gastrointestinal bleed within the past three months
  • Allergy to iodinated contrast media, antiplatelet drugs, heparin
  • Pregnancy
  • Life expectancy <12 months based on physician’s estimate

Data were collected from the electronic records and stored on a password protected Excel database (Microsoft Excel 2010, Redmond, WA, USA). Data on demographics, procedural aspects, technical success, patency rates and complications were documented. Longer-term patency rates (≥6 months) were not available at the time of writing the manuscript.

### Device

The Cleaner XT™ Rotational Thrombectomy System is percutaneous, 6Fr and catheter-based; it consists of a rotator drive unit attached to a sinuous-shaped radio-opaque wire. The drive unit rotates at approximately 4000 RPM, facilitating gentle mechanical declotting of occluded native vessel dialysis fistulae and synthetic dialysis access grafts. The Cleaner XT™ device was curated for smaller lumen vessels to allow easier manipulation and effective clot maceration.^11,12)^

### Procedure technique

**Figure 1:**
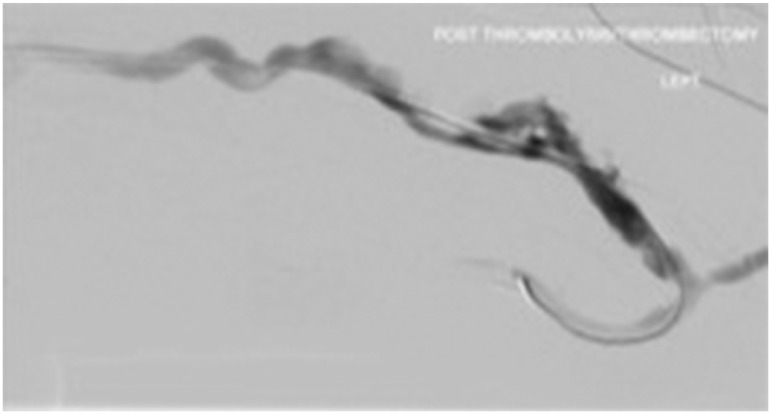
Fig. 1 Left brachiocephalic arteriovenous fistula after tissue plasminogen activator/angioplasty and trawling with Fogarty balloon, with residual thrombus trapped within the aneurysmal segment.

**Figure 2:**
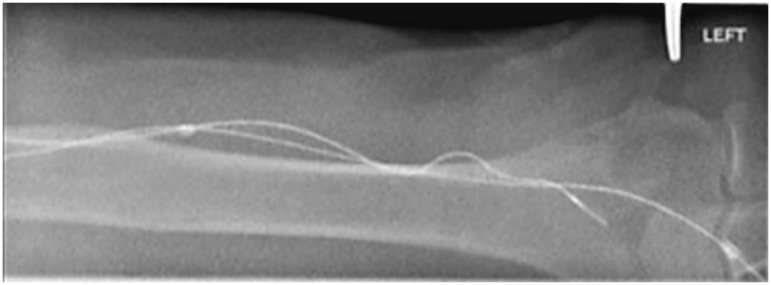
Fig. 2 Application of Cleaner XT™ in an aneurysmal segment of arteriovenous fistula.

**Figure 3:**
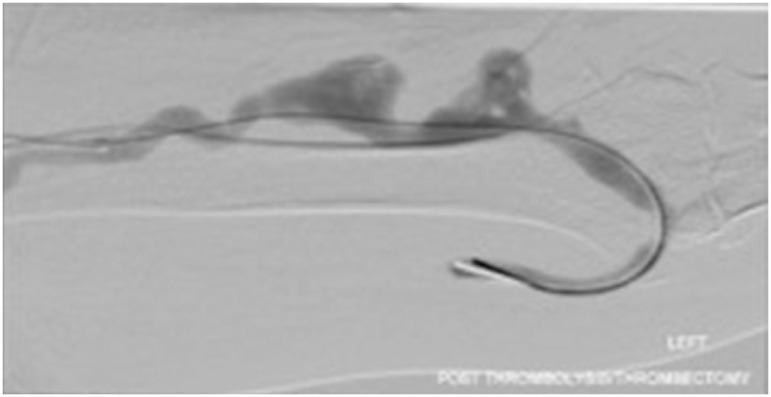
Fig. 3 The appearance of an aneurysmal segment of arteriovenous fistula after applying Cleaner XT™.

The procedure was performed according to our previously described centre protocol.^13,14)^ Briefly, antegrade and retrograde vascular sheaths/cannulae were placed in the thrombosed AVG/AVF under ultrasound guidance. The thrombolytic agent choices and doses were at the operator’s discretion. Via the sheaths/cannulae, 2–6 mg of recombinant tissue plasminogen activator (r-TPA) was instilled directly into the thrombosed segment of the access. When the operator chose urokinase, the occlusions were crossed with a glide wire and angiographic catheter antegradely. A central venogram was performed, followed by a pullback venogram. Urokinase diluted in 10,000 unit/mL concentration was then administered along with the thrombus via the angiographic catheter or thrombolytic catheter. Balloon angioplasty was then performed to treat the underlying culprit stenosis and macerate the clot. The arterial inflow was swept to clear the arterial plug at the arteriovenous or arterio-graft anastomosis using a 5.5Fr over-the-wire Fogarty balloon (**Fig. 1**). In the presence of residual thrombus, the Cleaner XT™ device was used for mechanical thrombectomy (**Fig. 2**). The device was introduced as many times as required to clear the residual clot (usually 2–3 times depending on the clot burden and adherence). The culprit stenotic lesions were either dilated with high-pressure balloons or stented, depending on recoil/dissection and location of the culprit lesions. Completion angiography was then performed to document flow restoration (**Fig. 3**).

### Definitions

The outcomes conformed to the practice guidelines published by the Society of Interventional Radiology (SIR).^15)^ Technical success was defined as restoration of flow with <30% residual stenosis on completion angiogram, while clinical success was considered the resumption of normal dialysis for at least one session. Procedural success was achieved if both criteria were met.

Primary patency was defined as the uninterrupted patency after intervention until the next access thrombosis and/or reintervention. Secondary patency was defined as patency after intervention until the access was surgically declotted, revised, or abandoned.

Complications were classified according to the American Society of Diagnostic and Interventional Nephrology system,^16)^ with the severity increasing from Grade 1 to Grade 4. Grade 1 complications were defined as those requiring nominal therapy, with an unplanned increase in the level of care but no adverse clinical consequences or sequelae. Grade 2 complications required minor treatment, such as percutaneous therapy, but were managed successfully with no significant chronic sequelae afterwards. The complications were also compared to the SIR classification,^17)^ where major complications included those requiring therapy and hospitalisation, permanent adverse sequelae and/or death and minor complications included overnight hospitalisation stay for observation only.

### Statistical analysis

Descriptive statistics are presented as proportions or means (± standard deviation) for categorical and continuous data, respectively. Comparison of technical and clinical success and primary and secondary patency rates was made by Fisher’s exact test. A p-value of <0.05 indicated a statistically significant difference. The analysis was performed using SPSS software (IBM, New York, NY, USA, v. 25, 2017).

## Results

A total of 17 procedures were performed on 17 patients. All patients had ESRD on haemodialysis. Baseline demographics are summarised in **Table 1**.

**Table 1:** Table 1 Baseline demographics

Characteristic				Number (%), mean (± SD) or median (range)
Total procedures				17
Gender		Male		8
		Female		9
Patient age (years)				66 (± 5.7)
Comorbidities	Diabetes mellitus			10 (58.8%)
	Ischaemic heart disease			7 (41.2%)
	Chronic lung disease			2 (11.8%)
	Cerebrovascular disease			3 (17.6%)
	Cancer			3 (17.6%)
Biochemical data on admission	Potassium			4.38 mmol/L (±0.479)
	Urea			19.0 mmol/L (±8.52)
	Creatinine			751 mmol/L (±286)
	Haemoglobin			11.2 g/dL (±1.42)
	Calcium			2.29 mmol/L (±0.13)
	Phosphate			1.52 mmol/L (±0.46)
	Albumin			36.3 g/L (±2.92)
HD access type	AVG			5
	AVF			12
Arteriovenous access age (days)	AVFs and AVGs			1425 (44 to 3488)
	AVFs			1838.5 (664 to 4404)
	AVGs			364 (33 to 1785)
Location of access	AVF		Brachiocephalic	10
			Brachio-basilic	1
			Radio-cephalic	1
	AVG		Brachio-axillary	1
			Brachiocephalic	1
			Brachio-basilic	3
Previous complications	Stenosis			9
	Haematoma			3
	Infection			1
	Aneurysm			1
Patients with previous intervention following access blockage prior to PMT				11
Time to intervention following access blockage				26 (6 to 151) hours
Local anaesthetic	1% lignocaine			15 (Mean 3.77 mL ±2.39)
	Not recorded			2
Location of blockage	Juxta-anastomotic segment			3
	Venous outflow			8
	Cephalic arch			5
	Intercannulation segment			4
	Vein-graft junction			2
Thrombolytic agent	Urokinase			7 (Median 90 000 U)
	Tissue plasminogen activator			9 (Median 4 mg)
	Not recorded			2
Complications	Grade 1			2
	Grade 2			2
Time to cannulation				0.353 (± 0.493) days
Post-operative antiplatelet/anticoagulation			Enoxaparin	6 (35.3%)
			DAPT	4 (23.5%)
			Aspirin only	1 (5.88%)
			Clopidogrel only	2 (11.8%)
			Warfarin	1 (5.88%)
			None	3 (2 failed procedures, 1 palliative care) (17.6%)

DAPT: dual antiplatelet therapy; SD: standard deviation; HD: haemodialysis; AVG: arteriovenous graft; AVF: arteriovenous fistula; PMT: pharmacomechanical thrombolysis

Both technical and clinical success were achieved in 88% (n=15) of the patients. Of the two unsuccessful cases, one was due to the patient’s inability to tolerate the procedure, so the patient underwent an AVF later. The other unsuccessful case is currently using a long-term haemodialysis catheter to create a new AVF.

There were no major perioperative complications. Vein rupture occurred in 18% (n=3) of the cases, but this did not involve the loss of the circuit. One of the patients became restless; thus, the procedure was abandoned in favour of creating a new AVF. The other two patients were treated initially with prolonged balloon tamponade; one case did not respond and required bailout stenting. These were classified as grade 2 complications. The other two cases of grade 1 complications were that of phlebitis and access site haematoma, which resolved with nominal therapy.

**Figure 4:**
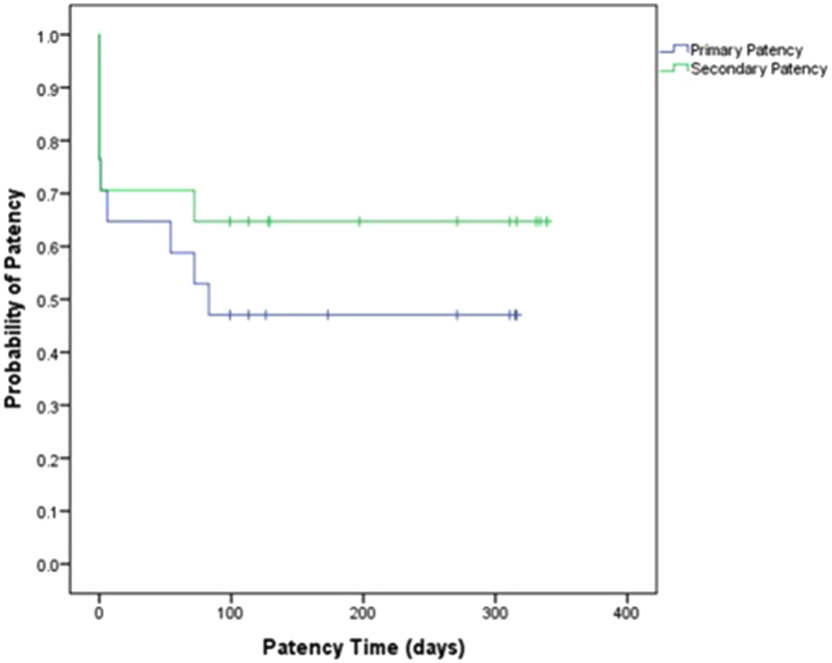
Fig. 4 Combined Kaplan–Meier curve for patency.

The mean primary patency time was 114 (± 116) days. There was a 65% one-month and 47% three-month primary patency rate, respectively, with an 83% one-month and 67% three-month primary patency rate in AVFs and a 20% 1-month and 0% three-month primary patency rate in AVGs. The mean secondary patency time was 155 (± 132) days, with a 76% one-month and 65% three-month secondary patency rate, respectively. There was an 83% one-month and three-month secondary patency rate in AVFs and a 60% one-month and 20% three-month secondary patency rate in AVGs. One of the patients who experienced technical and clinical success with this procedure subsequently opted for palliative treatment the day after the procedure(**Fig. 4**).

Two patients had more than 72 h time to intervention. When these were excluded from the analysis, there was a 73% one-month and 53% three-month primary patency rate, with an 83% one-month and 73% three-month primary patency rate in AVFs and a 25% one-month and 0% three-month primary patency rate in AVGs. There was an 87% one-month and 73% three-month secondary patency rate, respectively, with a 91% one-month and three-month secondary patency rate in AVFs and a 75% one-month and 25% three-month secondary patency rate in AVGs.

## Discussion

Pharmacomechanical thrombolysis is a relatively simple, minimally invasive procedure that can facilitate the rapid removal of clot under local anaesthesia. This potentially reduces surgical exposure, cost, treatment time, and intensive care unit stay compared to surgical thrombectomy. However, it also risks incomplete clot clearance and possible vessel injury.^5)^ In various studies, PMT has been shown to be an effective technique comparable to surgical thrombectomy in the treatment of thrombosis in arteriovenous fistulas and grafts with similar procedural success, patency, and complication rates^6,7)^ (**Table 2**).

**Table 2:** Table 2 Summary table of pharmacomechanical thrombolysis devices

Device	Mechanism of action	Advantages	Disadvantages	Access type (sample size)	Clinical success	Technical success	1 month patency rates			
							Primary	p-value	Secondary	p-value
Angiojet Rheolytic System (Yang et al., 2012)	- Based on Venturi effect by high-speed saline jets, thrombus is sucked into the device and macerated	- 360 suction vortex which theoretically reduces number of passes	- May leave residual thrombus adherent to wall	100% AVF (n=109) 33% upper arm	76%	77%	70%	0.78	76%	1
Arrow–Trerotola percutaneous thrombectomy device (Yang et al., 2012)	- Fragmentation of clot is done via generation of hydrodynamic vortex created by high-speed rotating impeller or basket	- Simple - Low manufacturing costs - Better contact with thrombus through mechanical action	- Causes significant endothelial denudation in native vessels	100% AVF (n=106) 38% upper arm	91%	91%	76%	0.38	90%	0.11
ClariVein catheter (Lim et al., 2017)	- Infusion of thrombolytic agents combined with a rotating catheter to augment the thrombolysis process	- Rapid rotational tip may allow cleaner removal of thrombus due to rheolytic effect of high-frequency spinning of eccentric tip	- Only studied in AVGs and not AVFs due to concern of endothelial injury from angulated tip - Rotating pin is smaller calibre and rotation speed is lesser as compared to Cleaner XT, hence may be less effective for large clots	100% AVG (n=11)	100%	100%	Not measured			
Cleaner XT	- Mechanical declotting of access via rotating mechanism	- May be more appropriate for AVFs - Curated for smaller lumen vessels to allow easier manipulation and effective clot maceration	- Limited data available so far	12 AVF, 5 AVG (n=17)	88%	88%	65%		76%	

PMT has also been shown to be comparable to CDT in the setting of deep vein thromboses in terms of patency rate, with a shorter length of stay leading to lesser costs.^8)^ For most studies, the drugs used in PMT are either TPA and/or urokinase. They are similarly effective in the pharmacomechanical thrombolysis of AVFs. However, the type of thrombolysis agent did not lead to a significant difference in primary or secondary patency rates.^13)^

A study comparing hydrodynamic recirculation devices (Angiojet rheolytic system, Boston Scientific, Marlborough, MA, USA) to rotational recirculation devices (Arrow–Trerotola percutaneous thrombectomy device, Teleflex, Wayne, PA, USA) showed a higher clinical success and secondary patency rate in the device with a rotational mechanism.^18)^ This might be due to the differences in the mechanism of action since the Venturi effect from hydrodynamic recirculation devices could potentially leave residual thrombus adherent to the wall. The percutaneous thrombectomy device had better contact with the thrombus through mechanical fragmentation and stripping, which resulted in a greater success rate. Examples of such devices include the ClariVein catheter,^19)^ the Arrow–Trerotola device,^6)^ and the Cleaner XT™ device.^20)^ The technical and clinical success rates for the former two devices were 100% and 91%, respectively, while the latter device has mostly only been reported in PMT of deep vein thrombosis. However, the ClariVein™ catheter has only been studied in haemodialysis grafts and not native AVFs, due to concern over endothelial injury from the angulated tip and spinning mechanism. This catheter was mainly designed for mechanochemical endovenous ablation of varicose veins^21)^ and was used as a novel method of PMT to unblock access grafts with a thrombolytic agent by Lim et al. in 2017.^19)^ The rotating pin is of smaller calibre (less than 3Fr versus the 6Fr catheter size of the Cleaner XT™ device) and the rotation speed is less (highest rate 3500RPM versus 4000RPM in the Cleaner XT™ device).^22)^ Both of these raise concerns that it may not be appropriate or effective in removing large clots from AVFs with a larger diameter.

In the study of the Arrow–Trerotola device, the difference in the success rate of the Arrow–Trerotola device was not significant when compared to our study’s success rate of 88% (p=0.67). The primary and secondary patency of the Arrow–Trerotola device at 1 month was 76% and 90%, respectively. This difference was not significantly different from our patency rates of 65% and 76% (p=0.38 and p=0.11, respectively). Other mechanical thrombectomy devices include the Trellis-8 catheter. This device’s use has not been described in terms of haemodialysis access in the current literature. However, success rates in arterial embolization^23)^ have been documented as 92%, which is not significant as compared to our rate of 88% (p=1.000). When used to treat deep vein thrombosis, grade II and III lysis was achieved in 93% of patients treated.^24)^ This rate is also comparable to our success rates in haemodialysis access (p=0.360). Further studies on rotational percutaneous thrombectomy devices have shown that the Cleaner device was associated with reduced endothelial damage as compared to the Arrow–Trerotola device in a rabbit model.^25)^ It is a promising option for treating deep vein thrombosis with pharmacomechanical thrombolysis.^20,26)^ There was also easy identification of stenosis regions as the normal sinusoidal pattern elongates in stenotic regions, helping to predict the need for balloon angioplasty even before angiography.^27)^ The comparable clinical success, patency and low complication rates reported herein also indicate that using the Cleaner XT™ device in thrombosed access grafts and fistulas is relatively safe and effective.

The KDOQI Vascular Access Guidelines^1)^ recommend that reasonable goals for graft patency would include a clinical success rate of >85% and three-month primary patency of 40%. In our study, the overall clinical success rate (88%) and three-month primary patency rate (65%) have met the KDOQI standards. Our study limitations include small sample size and a short follow-up period (at the time of data collection, most of the cases were less than one year post-procedure). A more extensive sample size and a longer follow-up time would be useful for further evaluation of the device as a PMT method. However, the immediate results of re-establishing flow and use of the access circuit for haemodialysis are excellent.

### Procedural tips and suggestions

The Cleaner XT™ wall contact thrombectomy device is especially useful during endovascular salvage of thrombosed AVF and AVG with aneurysmal segments because angioplasty balloons cannot make good contact with the vessel wall for clot maceration in the presence of aneurysmal dilatation. Significant residual thrombus frequently remains within the aneurysmal segment and will fall back into the vessel lumen after deflation of the balloons. It is critical to compress the aneurysmal segment with the fingers while applying the Cleaner XT™ device to allow good contact of the device with the residual thrombus in the aneurysmal segment. Alternatively, the aneurysmal segment can be compressed using an ultrasound probe to visualise the Cleaner XT™ device’s position within the segment. Since the Cleaner XT™ is not an over-the-wire device, supporting wires must be removed before applying the device. Therefore, adequate treatment of the culprit stenosis with re-establishment of partial blood flow is important before applying the Cleaner XT™. Any ruptures within the dialysis circuit should be excluded before applying the Cleaner XT™ device.

## Conclusion

The Cleaner XT™ for PMT is a feasible method of treating thrombosed haemodialysis fistulas and grafts. It has a relatively high technical and clinical success and low complication rate. However, a longer follow-up prospective study is required to assess its true efficacy.

## Additional Note

This study was accepted as a poster presentation at the Congress of the Asian Society for Vascular Surgery on 23 October 2019, Bali Nusa Dua Convention Center, Indonesia.
